# *Xanthomonas citri* subsp. *citri* and *Xanthomonas arboricola* pv. *pruni*: Comparative analysis of two pathogens producing similar symptoms in different host plants

**DOI:** 10.1371/journal.pone.0219797

**Published:** 2019-07-18

**Authors:** Jerson Garita-Cambronero, Marta Sena-Vélez, Elisa Ferragud, Pilar Sabuquillo, Cristina Redondo, Jaime Cubero

**Affiliations:** 1 Departamento de Protección Vegetal, Laboratorio Bacteriología, Instituto Nacional de Investigación y Tecnología Agraria (INIA), Madrid, Spain; 2 Centro de Investigación de Biocombustibles y Bioproductos, Instituto Tecnológico Agrario de Castilla y León (ITACyL), Villarejo de Órbigo, Leon, Spain; 3 Department of Biological Science, Florida State University, Tallahassee, Florida, United States of America; Universidad Nacional Autonoma de Mexico, MEXICO

## Abstract

Comparative studies in *Xanthomonas* have provided a vast amount of data that enabled to deepen in the knowledge of those factors associated with virulence and *Xanthomonas* plant interaction. The species of this genus present a wide range of host plants and a large number of studies have been focused to elucidate which mechanism are involved in this characteristic. In this study, comparative genomic and phenotypic analysis were performed between *X*. *citri* subsp. *citri* (*Xcc*), one of the most studied pathogens within *Xanthomonas*, and *X*. *arboricola* pv. *pruni* (*Xap*), a pathogen which has aroused great interest in recent time. The work was aimed to find those elements that contribute to their host divergence despite the convergence in the symptoms that each species cause on *Citrus* spp. and *Prunus* spp., respectively. This study reveals a set of genes that could be putatively associated with the adaptation of these pathogens to their hosts, being the most remarkable those involved in environmental sensing systems such as the case of the TonB-dependent transporters, the sensors of the two-component system and the methyl accepting chemotaxis proteins. Other important variants were found in processes related to the decomposition of the cell wall as could be appreciated by their dissimilar set of cell-wall degrading enzymes. Type three effectors, as one of the most important factors in delineating the host specificity in *Xanthomonas*, also showed a different array when comparing both species, being some of them unique to each pathogen. On the other hand, only small variations could be connected to other features such as the motility appendages and surface adhesion proteins, but these differences were accompanied by a dissimilar capacity to attach on host and non-host leaf surface. The molecular factors found in this work provide the basis to perform a more in-depth functional analyses that unveil those actual factors associated with pathogenesis and host specificity in *Xcc* and *Xap*.

## Introduction

*Xanthomonas* is a large genus of bacteria that encompasses species associated with plants causing serious economic losses in significant crops. Among the plant diseases due to xanthomonads, two are considered specially relevant in fruit trees, *Xanthomonas citri* subsp *citri* (*Xcc*), the causal agent of citrus bacterial canker (CBC) and *Xanthomonas arboricola* pv. pruni (*Xap*) that produces bacterial spot of stone fruits and almond (BSF) [1–3]. CBC is considered one of the major threat for citriculture and it is endemic in many citrus production zones, even though, *Xcc* is not present in other areas such as the Mediterranean basin or California in USA, where these bacteria are regulated as quarantinable [4]. BSF is worldwide distributed with a presence in most *Prunus*-growing regions, however *Xap* is still a quarantine organism in some areas like the European Union [5,6]. CBC is defined as a tropical or subtropical disease; meanwhile, BSF occurs mostly in temperate areas. *Xcc* and *Xap* are spread by wind-blown rains and may enter to the plant by injures or through the stomata, multiplying inside the tissue and being disseminated when proper humidity and temperature condition are present in the environment. Both, *Xcc* and *Xap*, cause lesions in leaf, fruit, stems and branches that visually may like quite similar between the two [2,3].

Studies to elucidate the mechanisms involved in the infection process have been conducted mainly in CBC but recently also in BSF, which has gained much more attention during the last few years with the prediction of putative virulence factors [1,7]. The availability of genomic information in *Xcc* and *Xap* has permitted the study of their secretion systems and effectors; particular attention has been paid on the type III secretion system (TSS3) and all its components that play a major role in virulence. Moreover, other factors involved in the different stages of the infection have been separately studied or predicted in both species [7–9].

One of the most intriguing topic in *Xanthomonas* genus is the high degree specialization of the species or even the host specificity within the species that may comprise multiple pathovars [10–13]. The reason why some members of *Xanthomonas* are able to infect specifically a host and no others, and why close genetically strains or species long differ in the host, is constantly under debate but not completely solved [12,14,15]. Comparative analyses on the genomes of *Xcc* and *Xanthomonas campestris* gave some clues on host specificities at the beginning of the omic era [16]. Moreover, either in *Citrus* or in *Prunus* species, the pathogenic strains of *Xcc* and *Xap* may coexist with other xanthomonads, less o non-aggressive, that produce no severe symptoms or no symptoms at all. The comparison with those strains in the same host has helped to identify some factors involved in pathogenicity in these two models [8,17]. Herein, we have been going further and compared two particular xanthomonads models that have in common their availability to infect woody plants producing similar symptoms but differing in their host species and environmental conditions. The goal was to get an idea of the common mechanisms that underline the infections to produce similar effects in their different hosts and try to elucidate those that may contribute for the host selection. Comparative genomic analysis has been made based on data available in databases but also increasing the number of *Xap* genomes by obtaining two from strains that showed different motility features. The work was aimed to identify those genes specifically absent or present in each bacterial species and try to associate the differential genome content to phenotypic characters that could play a role predominantly in the early stages of the virulence process.

## Results

### Genome sequencing of *X*. *arboricola* pv. *pruni* strains CITA 9 and CITA 99

To expand the available genomes batch of *X*. *arboricola* pv. *pruni*, whole genome sequences of *X*. *arboricola* pv. *pruni* strains CITA 9 and CITA 99 were obtained (Table 1). These strains were selected based on their motility on semisolid surfaces and ability to cause disease on almond, peach and plum [8].

**Table 1:** *Xanthomonas* strains used in this study.

Strain	Specie	Host	Origin	Reference
CITA 9	*X*. *arboricola* pv. *pruni*	*Prunus persica*	Spain	This study
CITA 99	*X*. *arboricola* pv. *pruni*	*Prunus amygdalus*	Spain	This study
*Xcc* 306	*X*. *citri* subsp. *citri*	*Citrus* sp.	Brazil	[16]
CITA 33	*X*. *arboricola* pv. *pruni*	*Prunus amygdalus*	Spain	[18]

Main genomic features of CITA 9 and CITA 99 are shown in Table 2. A total of 41,846,862 reads were obtained from the sequencing of CITA 9, while 26,414,106 reads were obtained after sequencing of CITA 99. These reads were assembled in a draft genomes of 5,061,994 bp and 5,075,205 bp for CITA 9 (fold coverage 96x, 57 contigs with a N_50_ of 148,657 bp) and CITA 99 (fold coverage 97x, 60 contigs with N_50_ 147,514 bp), respectively. Automatic annotation of the genome sequences, with the NCBI Prokaryotic Genome Annotation Pipeline, found a total of 4,445 total coding sequences, 4,152 protein coding sequences, 5 complete rRNAs [5S (2), 16S (1), 23S (2)] and 51 tRNAs for the draft genome sequence of CITA 9, meanwhile CITA 99 showed a total of 4,431 coding sequences with 4,147 protein coding sequences, 5 complete rRNAs [5S (3), 16S (1), 23S (1)] and 51 tRNAs. In addition, complete genome sequence of the potential virulence associated plasmid p*Xap*41 [19] was found in the draft genome sequence of both strains (Table 2).

**Table 2:** Genome sequence information and statistics of *X*. *arboricola* pv. *pruni* strains CITA 9 and CITA 99.

Property/Attribute	CITA 9	CITA 99
	Value	Value
Strain synonyms	-	IVIA 3161-2-1; CFBP 7100
Sequencing platform	Illumina HiSeq platform	Illumina HiSeq platform
Fold coverage	96x	97x
Assemblers	CLC and MIRA	CLA and MIRA
Genome annotation	NCBI-PGAP	NCBI-PGAP
Locus tag	EJK96	EJL05
GenBank ID	RWYS00000000	RWYT00000000
Genome size (bp)	5,061,994	5,075,205
DNA G+C (%)	65.4	65.4
Total genes	4,539	4,525
Protein coding sequences	4,445	4,431
RNA genes	94	94
Genes with function prediction	3,651	3,633
Genes assigned to COGs	2,184	2,181
Genes with Pfam domains	2,247	2,241
Genes with signal peptides	612	611
Genes with transmembrane helices	970	975
CRISPR repeat unit	0	0
Plasmids	1 (p*Xap*41)	1 (p*Xap*41)

Average nucleotide identity (ANI) among the genome sequence from the two strains, obtained here, and the strains of *Xap* available in databases, was over 99%, confirming all of them as part of the same bacterial species (S1 Table).

### Comparative genomics of *X*. *arboricola* pv. *pruni* and *X*. *citri* subsp. *citri*

**Fig 1:**
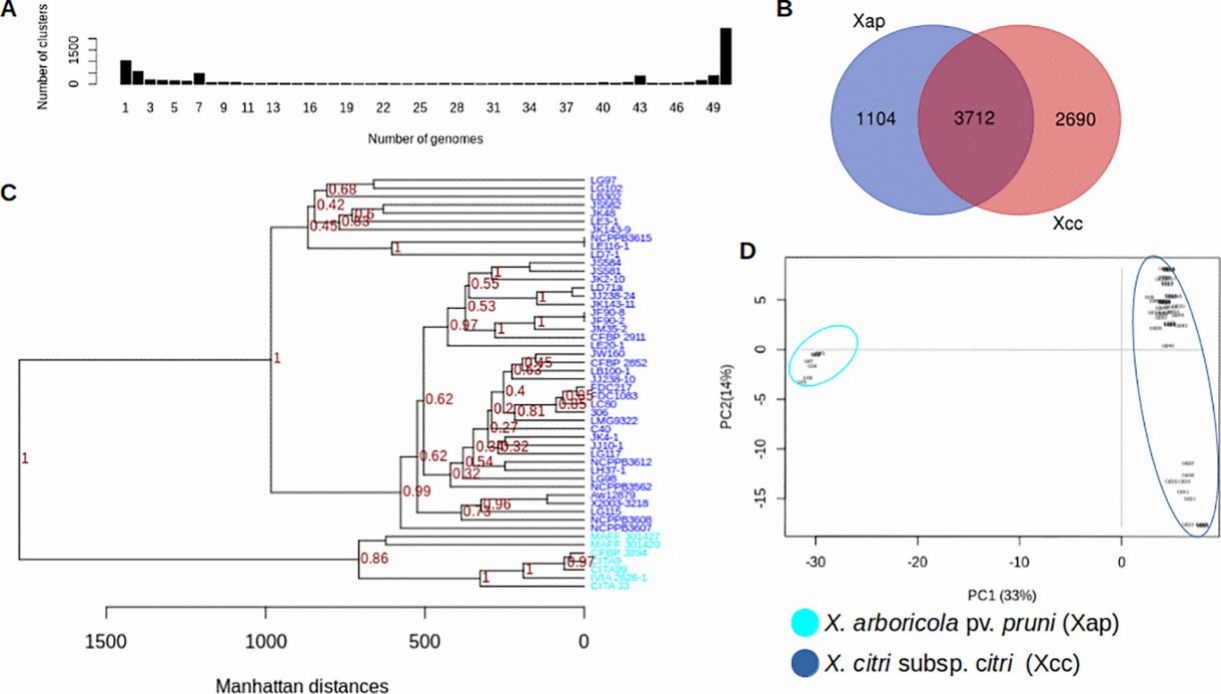
Genome comparative analysis between *Xanthomonas arboricola* pv. *pruni* (*Xap*) and *X*. *citri* subsp. *citri* (*Xcc*). (A) Distribution of unique gene clusters per genome sequence. (B) Gene clusters shared and unique to each *Xanthomonas* species. (C) Cluster comparative analysis (bootstrap values showed at the branch points) and (D) principal component analysis spanned by the two principal components based on the presence/absence of 7,506 gene clusters in the 50 genome sequences of *Xap* and *Xcc*.

Genome comparative analysis of seven *Xap* and 43 *Xcc* strains (S1 Table), permitted to find a total of 222,751 protein coding sequences that were clustered, according to pairwise blast comparisons, in 7,506 gene clusters. Based on a gene/genome distribution previously proposed [20], these genes were distributed as follows: 2,447 genes were shared by 100% of the strains, 614 genes were present in 95%, 2,184 were present in the genome sequence of at least 15% of the strains and 2,127 were present in less than 15% of the strains; from these “cloud genes”, 1,036 were found only in one single genome (Fig 1A).

**Fig 2:**
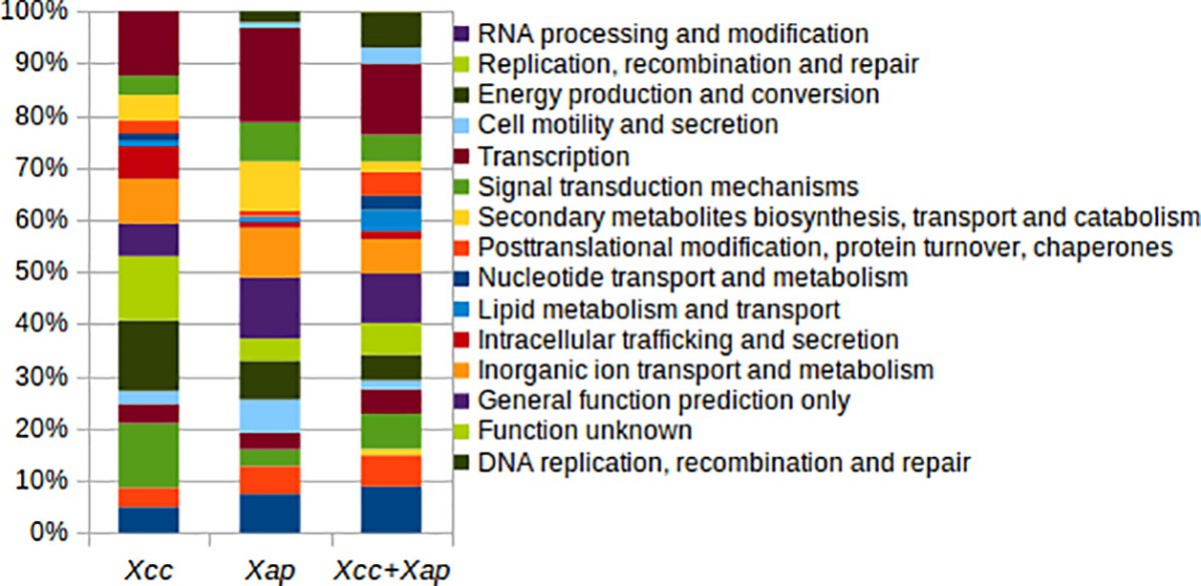
Relative frequency distribution of the unique gene clusters found in the genome sequences of seven strains of *X*. *arboricola* pv. p*runi* (*Xap*) and 43 strains of *X*. *citri* subsp. c*itri* (*Xcc*) as well as those gene clusters shared by both bacterial species (*Xcc + Xap*). Only those gene clusters that were complete according to the annotation obtained from the COG database were counted and represented (81, 92 and 2,402 protein sequences for *Xap*, *Xcc* and *Xcc+Xap* respectively).

From the entire orthologous gene clusters found, 3,712 were shared by *Xap* and *Xcc* strains; 1,104 were unique to *Xap* and 2,690 were only found in the genome sequences of *Xcc* (Fig 1B). Representative sequences of each one of the gene clusters, were classified into general protein functions according to the COG database; as a result, from the 3,712 shared by both species, only 2,402 corresponded to complete gene sequences of the COG database (without any truncation in the N or C protein terminus) and from these, 2,033 had a COG function assigned being those associated with the transcription process (13,6%) the most abundant (Fig 2).

A high number of the specific sequences for *Xap* and *Xcc* were annotated as hypothetical proteins according to the NCBI database. Taking into account only those complete protein sequences with a COG functional annotation (81 and 92 protein sequences in *Xap* and *Xcc*, respectively), they were mostly identified as proteins with a transcriptional function (12% and 18% in *Xap* and *Xcc* respectively) (Fig 2). According to the annotation of the unique protein sequences of each species, those 23 with a potential virulence function in *Xap* consisted in eleven annotated as TBDTs (sequences ID at NCBI database: WP_052152606.1, GAE49251.1, GAE59124.1, GAE52322.1, KCX01026.1, KCW99720.1, KPN11333.1, KCW99074.1, KCW99075.1, WP_081327680.1, KCW98622.1), one MCP (KCW98291.1), one cellulase (GAE53805.1), two lipases (DK27_22630, KCW98617), one xylanase (KCX01616.1), three components of the T3SS (KCX00174.1, GAE61338.1, GAE53745.1) and four potential effectors (GAE53798.1, KPN11355.1, KCX00187.1, KCW99719.1). On the other hand, in *Xcc*, it was found 28 putative virulence protein sequences comprising four TBDTs (AAM38897.1, AGI07591.1, AAM35882.1, AAM35579.1), six STCRs (AAM37649.1, AAM37020.1, AAM36995.1, AAM36994.1; AGI08218.1, AAM3798.1), one MCP (AGI08281), one adhesin (AAM38948.1), one cellulase (AGI09979.1), nine components of a T4SS (AAM39283.1, AAM39284.1, AAM39285.1, AAM39286.1, AAM29287.1, AAM39290.1, AAM39291.1, AAM39292.1, AAM39293.1), two components of the T3SS (AAM35284.1, AAM35307.1) and four effector proteins (AGI06427.1, AGI10546.1, CEH61339.1, AAM35178.1,). Finally, when analyzing the similarity of the 50 bacterial strains based on the distribution of each one of the gene clusters in their genome sequences, both species were clearly separated as two different taxa as represented by the similarity tree and the distribution of the strains in the space according to the two most important components on the principal component analysis (Fig 1C and 1D). These results have been also confirmed by the average ANI values observed. Within species ANI values over 99% were shown, meanwhile ANI average value among genome sequences of *Xap* and *Xcc* was below 90% (S1 Table).

In addition to the potentially virulence-associated genes found in each one of the analyzed species, a set of 410 genes previously described in *Xanthomonas*, related to the whole disease process were searched in all the 50 strains selected by using the BLAST algorithm. Tabular BLAST results were filtered and only those gene sequence alignmets with a percentage of identical matches (identity) and a percentage of aligned sequence length (query coverage) over 80% were considered as present in each one of the 50 genomes. The distribution of those genes found in *Xap* and *Xcc* strains by the abovementioned approach is described below.

### Comparative analysis based on putative virulence-associated genes in *Xanthomonas* spp

#### a) Surface structure and appendages

Infection in CBC or BSF seems to be started by bacterial attachment and colonization of host tissues through surface structures and appendages. Moreover, prominent roles of motility and chemotaxis were identified at the beginning of the genomic studies in *Xanthomonas* [16]. Both species present a total of thirty orthologs that conformed the complex of genes involved in flagella and its regulation and no differences were shown between *Xap* and *Xcc*. In addition, *Xap* and *Xcc* include genes for type IV fimbriae (pili) and fimbrial adhesins, however *Xcc* showed thirty one of them, meanwhile from *Xap*, only twenty genes could be identified, all of them in common with *Xcc*. The eleven genes specifically found in *Xcc* were identified as *pil* or *fim* genes. Moreover, four genes related to non fimbrial adhesins are shared between both groups, showing *Xcc* an additional specific protein (homolog to XCV2672). Therefore, although both type of strains contain mostly similar structures for adhesion to plant surface, minimum differences on adhesions may give rise for a specific interaction with the host.

#### b) Environmental sensing and response

Environmental sensing by bacteria is also a main factor affecting the virulence and bacterial behavior and may play a role on the host selection. Herein, a comparison was made on environmental sensors used by the bacteria to response to the different surrounding conditions.

Five genes for TBDTs are shared between *Xap* and *Xcc* but three more were identified as specific in *Xap* (XCC1719, XCC3595 and XCC4162) and six in *Xcc* (XAC0291, XAC0852, XAC2185, XAC2193, XAC3050 and XAC4062). The orthologue to XAC3050, which corresponds to a TonB receptor, was identified in 100% of the *Xcc* strains analyzed but it was not found in any of *Xap* strains.

A large repertoire of STCRs was found either in *Xap* or *Xcc*. This repertoire involves fifty two genes shared by the strains of both species and seventeen specific ones for *Xcc* (XAC0326, XAC2192, XCV3165, XCV2623, XAC0136, XAC1282, XAC3643, XAC1075, XAC2167, XCV2111, XAC1819, XAC2804, XCV3166, XCV4223, XCV2105, XOO3875, XCV3267).

Different batches of MCPs were identified in both groups of strains. Twenty MCPs are mutual and were found at least in one strain of each species; two class I MCPs receptors are present only in *Xap* (homologs to XCC0276 and XCC0324) and other three are specific of *Xcc* (homologs to XCV1938, XCV1939, XCV1942). Besides, seven of the twenty three MCPs identified in *Xcc*, are present in all the strains of this group, meanwhile only one (homolog to XCV1778), out of the twenty two identified in *Xap*, is common to every strain. Moreover, this gene has been also identified in 100% of the *Xcc* strains, indicating a putative and essential common role in both species.

**Fig 3:**
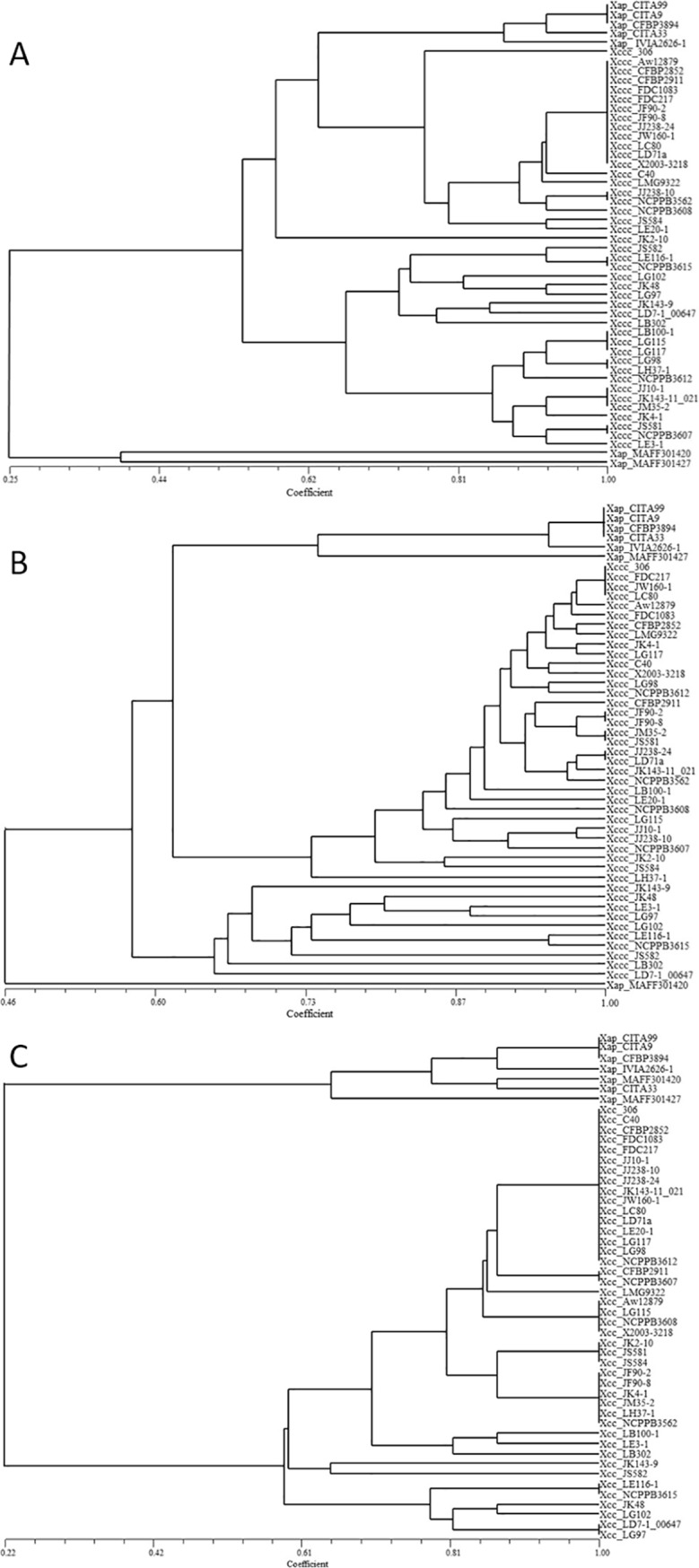
**Dendrogram based on the profiles for methyl accepting chemotaxis proteins (MCPs) (A), sensors of the two-component regulatory system (STCRs) (B) and TonB- dependent transporters (TBDT) (C) in *Xanthomonas citri* subsp. *citri* (*Xcc*) and *Xanthomonas arboricola* pv. *pruni* (*Xap*).** Similarities were calculated, based on sensor presence/absence data converted to a binary form, according to the Jaccard´s coefficient and clustering achieved by UPGMA using the NTSYS version 2.1.

To reveal if the environmental sensors analyzed play a role in the host specificity of *Xap* and *Xcc*, their profiles were analyzed by a similarity and cluster analysis based in presence/absence of the gene sensors and according to Jaccard´s coefficient (Fig 3). This analysis revealed two main major clusters that correlated with the two *Xanthomonas* species evaluated, however particular profiles were shown in some cases for strains of *Xap* isolated from Japan (MAFF 302420 and MAFF 301427). The mean level of similarity between main clusters corresponding to *Xap* and *Xcc* were 24.6±7.1% for TBDTs, 59.8±7.1% for STCRs and 56.6±9.8 for MCPs. Meanwhile, similarities within *Xcc* and *Xap* groups were for TBDTS of 85.0±12.05 and 76.2±16.0, for STCR of 98.0±2.6 and 76.3±15.0 and for MCPs, 92.5±6.0 and 68.0±17.8, respectively. The most apparent difference between *Xap* and *Xcc* was in TBDTs profiles, which may indicate a major role of these structures in their plant host differentiation.

*Xanthomonas* uses a cell-to-cell communication or ‘quorum sensing’ (QS) system for promoting collective behaviors which coordinate the expression of a multitude of genes and this depends of the diffusible signal factor (DSF). *Xap* and *Xcc* contain the ten genes involved in synthesis, perception of the DFS and transduction of the signal, although *Xap* presented an additional gene (*rpfl*) that is related to its regulation (analogue to XCC1847).

#### c) Type secretion systems II and III

Both species of *Xanthomonas* present genes associated with functional T2SS and T3SS and therefore the mechanisms to secrete a battery of degrading enzymes and virulence effectors.

*Xcc* and *Xap* share 13 genes related to the T2SS machinery but showing in addition two genes only found in *Xap* (XCC0664 and XCC3419), corresponding to genes *xcsJ* and *xpsI*, associated to pseudopilins and maybe related to the secretion of different substrates [21].

On the T3SS, *Xap* and *Xcc* share 17 genes of *hrp* (hypersensitive response and pathogenicity) gene cluster. However, *Xcc* showed additional eleven genes *hrc* (*hrp*-conserved) or *hpa* (*hrp*-associated), some of them related to putative chaperones that may assist the effector secretion (S2 Table).

#### d) Degradative enzymes

Plant cell wall is one of the main barriers between the host plant and the plant pathogen. As most of the phytopathogenic bacteria, *Xap* and *Xcc* are extracellular parasites that occupy intercellular spaces and need to decompose the surrounding cell-walls mainly composed by pectin and cellulose. *Xcc* and *Xap* have a repertoire of genes that codify for enzymes with cellulolytic, hemilcellulolytic and pectolytic activities. In case of cellulolytic enzymes, in *Xap*, twelve genes for cell wall degradation were found, eleven of them are shared between *Xcc* and *Xap*, but one specific gene was identified with this activity in each group, XCC3881 in *Xap* and XAC3516 in *Xcc*. Seven orthologues for pectolytic enzymes were found in both species, four of them are shared by both species and two enzymes were identified specifically in *Xcc* (XCC0644 and XCC0645) and one in *Xap* (XCC3459). Moreover, a total of eleven common orthologs were found for hemilcellulolytic enzymes in both bacterial species.

#### e) Type III effectors and other Type III secreted proteins

**Fig 4:**
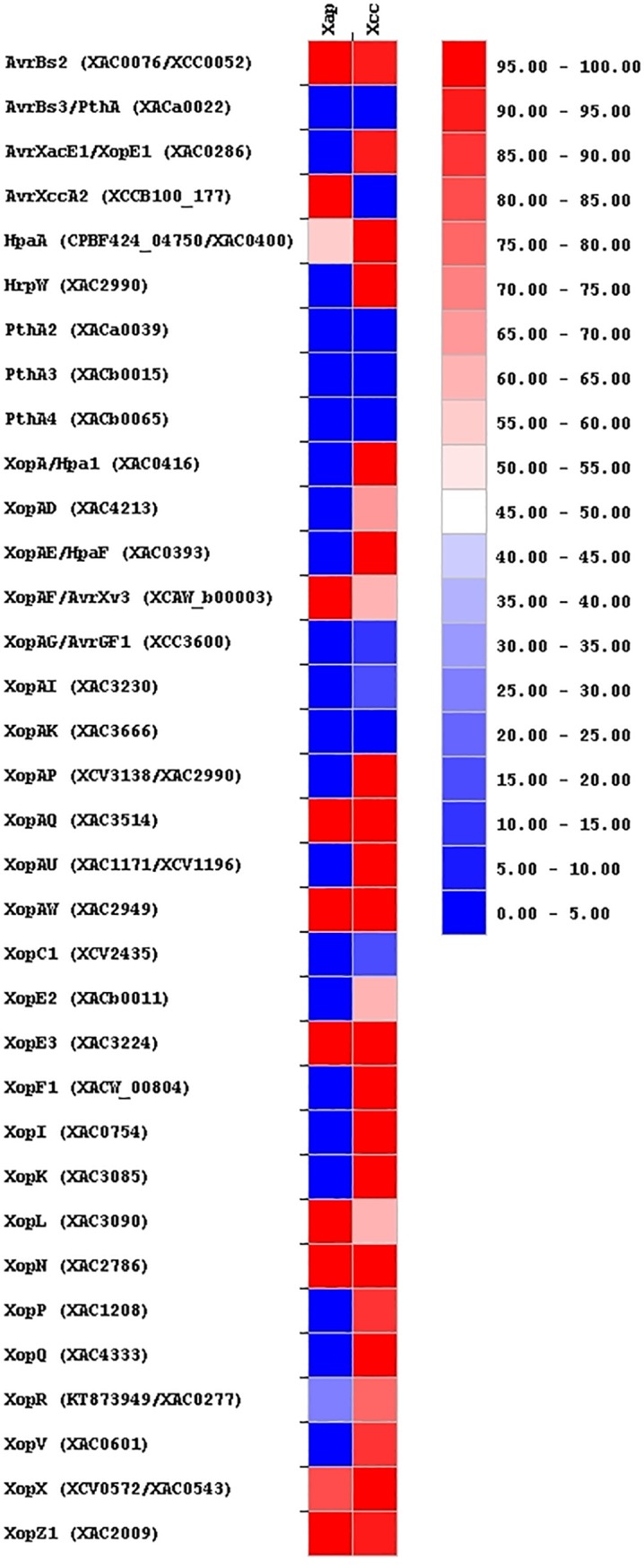
Distribution of the type III effectors (T3Es) and other type III secreted proteins (T3SPs) among the bacterial strains of *X*. *arboricola* pv. *pruni* (*Xap*, 7 strains) and *X*. *citri* subsp. *citri* (*Xcc*, 43 strains). Color scale indicates the percentage of strains containing each one of the searched proteins.

Due to the importance of the T3SS on the *Xanthomonas* [14], the profile of T3Es and other Type III secreted proteins (T3SPs) of *Xap* and *Xcc* was also predicted. *Xcc* showed a larger repertoire conformed by 27 T3Es and 2 T3SPs [HpaA (XAC0400) and HrpW (XAC2990)] (Fig 4). From these, 10 [XopA/Hpa1 (XAC0416), XopAP (XCV3138/XAC2990), XopAU (XAC1171/XCV1196), XopAW (XAC2949), XopE3 (XAC3224), XopI (XAC0754), XopK (XAC3085), XopN (XAC2786), XopQ (XAC4333) and XopX (XCV0572/XAC0543)] were found in all the 43 strains analyzed. On the other hand, 12 T3Es were found in the genome sequences of *Xap* strains (Fig 4); from these, nine [AvrBs2 (XAC0076/*XCC*0052), Avr*Xcc*A2 (*XCC*B100_177), XopAF/AvrXv3 (XCAW_b00003), XopAQ (XAC3514), XopAW (XAC2949), XopE3 (XAC3224), XopL (XAC3090), XopN (XAC2786), XopZ1 (XAC2009)] were present in all the genome sequences of this species. From the T3Es present in *Xap* only Avr*Xcc*A2 was unique in this species. Sequences associated with other 10 T3Es (XopAF, XopAH, XopAI, XopAK, XopQ, XopE2, XopF1, XopG, XopQ and XopV) and to the T3SPs HrpW and HpaA were found in *Xap* strains but they seem to be incomplete, truncated or showed a percentage of coverage or identity lower than 80% when comparing with those genes listed in S2 Table. Therefore, in order to perform the comparative analyses between both species, they were considered as completely absent in *Xap*. Finally, those effectors determined as TAL effectors [AvrBs3/PthA (XACa0022), PthA2 (XACa0039), PthA3 (XACb0015), PthA4 (XACb0065)] were only found in some strains of *Xcc* (Fig 4).

### Biofilm on abiotic surfaces

**Fig 5:**
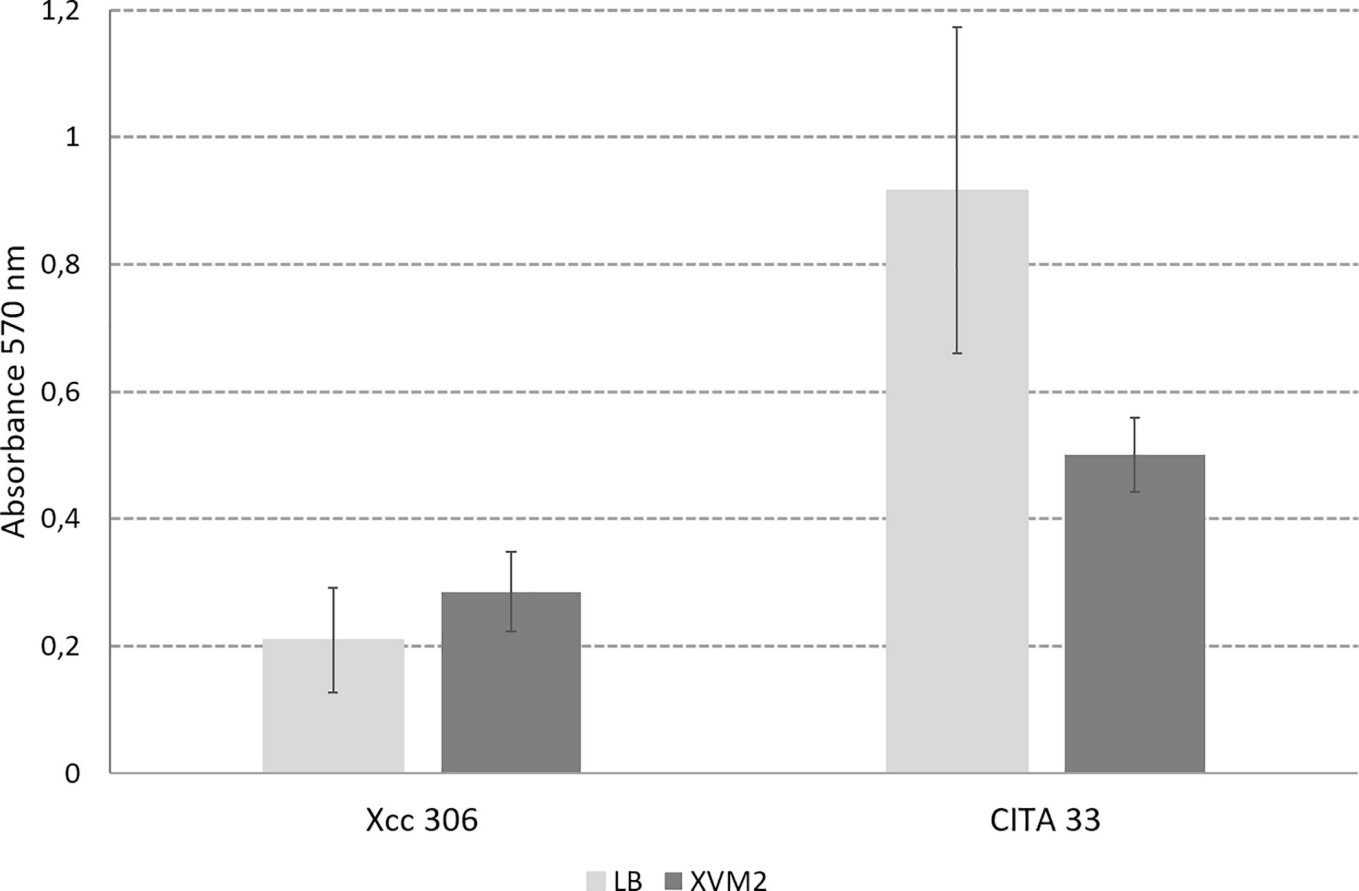
Biofilm formation by *Xanthomonas citri* subsp. *citri strain* 306 and *Xanthomonas arboricola* pv. *pruni* strain CITA 33 on polypropylene surface quantified by absorbance of crystal violet stain. Data were analyzed as shown in the main text.

*Xcc* and *Xap* formed biofilms when they were cultured in either LB, a nutrient rich broth, or XVM2, which simulates apoplast condition and induces virulence factors (Fig 5). When compared to *Xcc* strain 306, *Xap* strain CITA 33 seemed to produce larger amount of biofilm. In addition, *Xap* strain formed significantly (P<0.05) more biofilm when grown in LB medium than in XVM2 medium, meanwhile *Xcc* strain showed the opposite behavior revealing more aggregation in the apoplastic simulated condition which involves nutrient limitation and lower bacterial growth.

### Biofilm on biotic surfaces

Bacterial aggregation on biotic leaf surfaces was also observed in both *Xanthomonas* strains evaluated. Moreover, our results revealed that it occurred in all the plant species analyzed, host or non-host, but with different aggregation patterns. For each plant pathogen bacterial strain, distribution pattern was different according to the incubation period tested.

**Fig 6:**
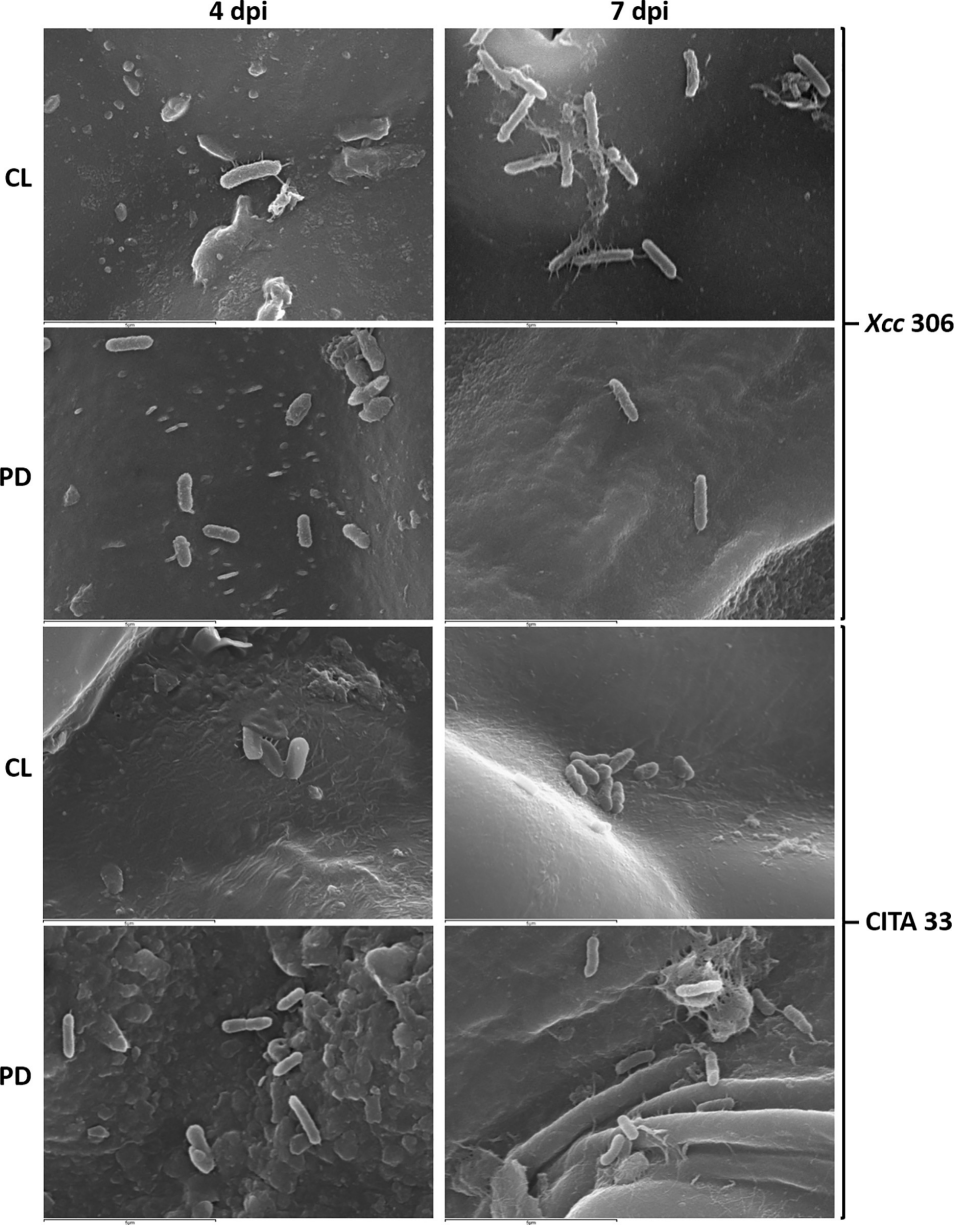
Scanning electron microscopy of *Xanthomonas citri* subsp. *citri* strain 306 and *Xanthomonas arboricola* pv. *pruni* strain CITA 33 onto the leaf surface of *Citrus lemon* (CL) and *Prunus dulcis* (PD) after 4 and 7 dpi. Scale bar 5 μm.

*Xcc* strain 306, a natural pathogen of lemon, was able to establish fibers connecting the bacterial cell with the plant epidermis at 4 dpi (days post inoculation). After 7 dpi this strain showed developed structures not only connected to the leaf surface but also forming well defined aggregates. When *Xcc* 306 was inoculated onto *P*. *dulcis*, no bacterial aggregates were detected at 4 or 7 dpi; however it was able to produce lateral adhesion filaments attached to the plant surface after 7 dpi. *Citrus lemon* leaves were susceptible to be colonized by *Xap* strain CITA 33 and some bacterial aggregates were observed. Furthermore, at 7dpi the presence of bacterial cells of CITA 33 was confirmed on the leaf surface of citrus species and a biofilm mature structure was observed (Fig 6).

**Fig 7:**
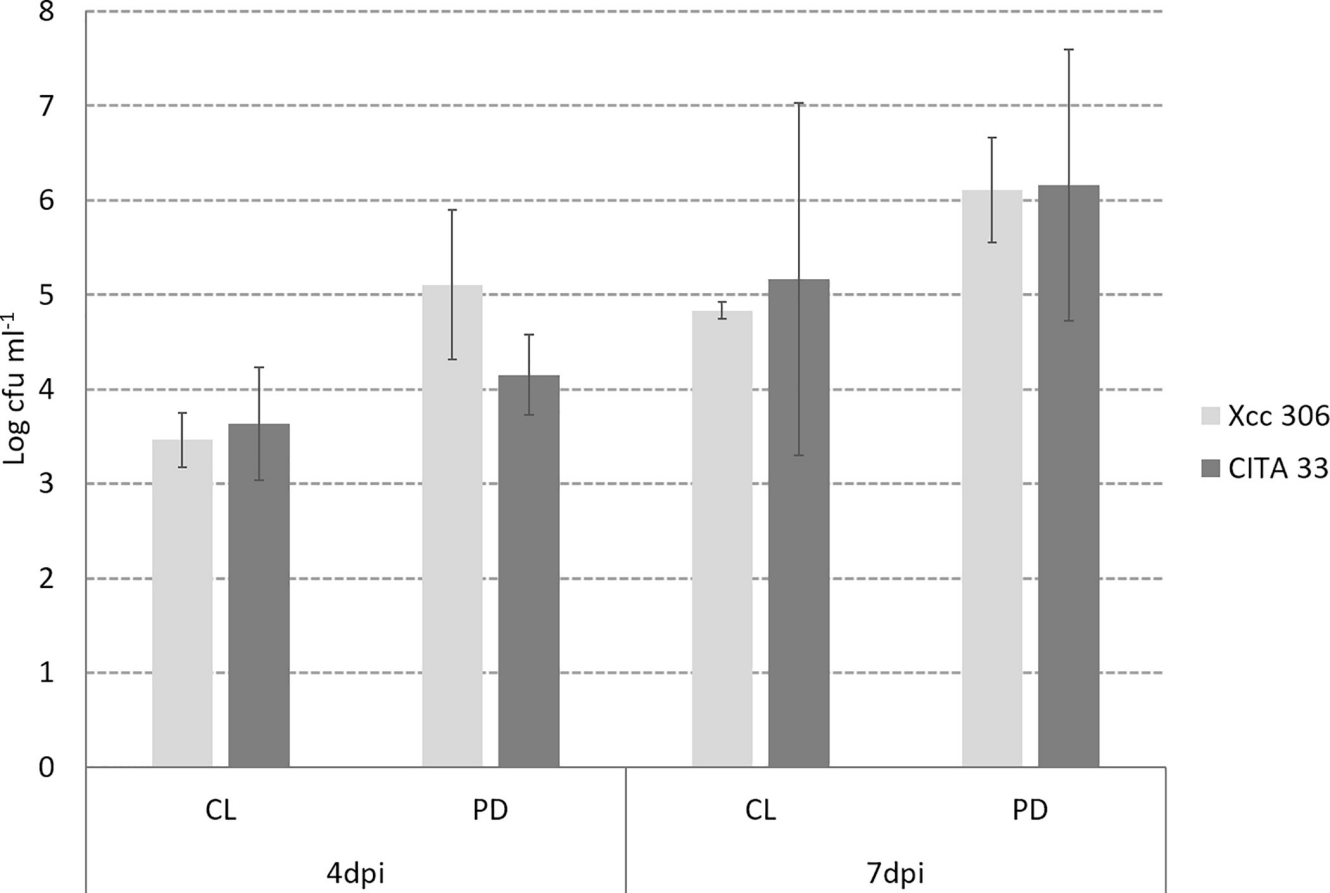
Survival of *Xanthomonas citri* subsp. citri strain 306 and *Xanthomonas arboricola* pv. *pruni* strain CITA 33 onto the leaf surface of *C*. *lemon* (CL) and *P*. *dulcis* (PD) quantified by isolation and serial dilutions at 4 and 7 dpi. Mean from three replicates with the standard deviation are represented in each bar.

Bacterial survival onto leaf surfaces seemed to be more related with the host than with the bacterial species inoculated onto the surface (Fig 7). No differences in survival were shown between both species on the different hosts, however the number of culturable cells for both strains was higher on almond compared to lemon leaves.

## Discussion

This work was aimed to improve the knowledge of the possible mechanisms involved in the infection of two *Xanthomonas* species and to determine those that may play a key role in host range. *Xcc* and *Xap* produce similar symptoms in their hosts, which are in both cases woody plants. Moreover, the disease cycles for citrus bacterial canker and bacterial spot of stone fruits, seem to be analogous and may incorporate an epiphytic phase at early stage of the infection. Although biofilm formation is not an essential step in CBC, *Xcc* may form organized bacterial aggregates that facilitate the disease progress and contribute to the bacterial survival and virulence [22–25]. Furthermore, *Xap* multiplies epiphytically on leaf forming biofilm structures that may assist in the infection development [2]. In addition, both, *Xap* and *Xcc*, contain secretion systems to discharge virulence factors that trigger the infection processes in later stages [1,2].

Since the beginning of the “omics” studies in *Xanthomonas* [16], comparative analyses within and among *Xanthomonas* species, have facilitated our understanding of the pathogenic processes as well as unveiled those molecular factors involved in virulence and host range determinacy [10,26].

In this work, first, a comparative genomic analysis of complete genomes available for *Xap* and *Xcc* was performed in order to elucidate general differences between the two bacterial groups. Unsurprising, average nucleotide identities among *Xap* and *Xcc* strains was below 90%, meanwhile the same within the species was around 99%, confirming the taxonomic different adscription of such species inside the *Xanthomonas* genus according to standards [27,28]. However, 77% of the orthologous gene clusters found in *Xap* were shared with *Xcc*, and in *Xcc*, 58% of them were shared with *Xap*. Therefore 23% and 42% of the gene clusters were unique for each species.

Herein, genes involved in surface structure and appendages were identified in *Xap* and *Xcc*. Both bacteria contain mostly similar structures, for instance, they enclose the same flagella machinery, although motility described in the two models is not identical. Even though swimming motility has been confirmed in *Xcc* and *Xap* [8,25], surface motility appeared to be different between them. *Xap* seems to present typical swarming flagella-dependent motility and some of the strains show dendritic-like patterns [8], meanwhile, *Xcc* can spread on semisolid agar surfaces by way of sliding motility, a mechanism independent of flagella [23]. In addition, both species showed genes related to the Type IV pilus that is associated to twitching motility [29,30] and it was described either in *Xap* or *Xcc* [8,31,32]. Nevertheless, differences were identified in *pil* and *fim* genes involved on pili or fimbria structure. Fimbria have been described in different *Xanthomonas* to be involved in early stage of the infection mediating specific adherence to the host [33]. Our results on adhesion to host and non-host leaf surface are congruent with this genomic difference, presenting dissimilar aggregation patterns when these species were or not inoculated on their plant host surface. However, these species survive similarly on leaf regardless the host in the line of results recently published [34]. This might be due to the short time incubation period evaluated that made impossible to determine the actual survival ability of the bacteria, or because experimental conditions were artificially favorable for them, resulting in an environment where their survival structures were not needed for population persistence. As emphasized in previous work, further investigation is needed to determine the effect of the environmental conditions for the bacterial survival on non-host surface [34]

Plant pathogenic bacteria are able to sense and respond to their environment and this is a crucial step at early stages of the interaction with the plant. Variation in environmental sensors has been observed between *Xap* and *Xcc*, where large repertories of TBDTs, STCRs and MCPs were identified. Considering TBDTs, *Xap* and *Xcc* showed a dissimilar repertoire sharing only five of them. In addition to the set of TBDTs previously known in *Xanthomonas*, a group of new genes annotated as TBDTs, which are unique to each one of the species studied in this work, have been identified, increasing the number of sensing proteins that differentiate both species. In summary, taking into account all TBDTs identified, *Xap* and *Xcc* contained a total of 14 and 10 unique TBDTs, respectively. It should be noted that TBDTs have been associated with different infection mechanisms such as biofilm formation, transition to sessile lifestyle on *Xcc* [35–37] or assimilation of the substrates provided by the host plants in *Xanthomonas* [15], all of them connected to host range.

Variable responses to the environmental conditions have been observed in both species in previous studies. In *Xcc*, chemotaxis, mediated by MCPs, was indicated to be fundamental to the process of colonization of plant tissue during the disease induction [38], meanwhile strains of *Xcc* and *Xap* from different hosts, show a variable response to certain synthetic compounds and also distinct MCPs profiles [8, 39]. The response to the environmental composition and its posterior motility towards or against the chemical present is directed by MCPs, whose profile could be associated with the host specificity, as well as with the coevolution developed during the plant-microbe interactions [37]. As occurred with TBDTs and STCRs, most MCPs are shared by *Xap* and *Xcc*, although some differences could be elucidated. For instance, orthologs of MCP XCC0324 were specifically identified in *Xap*, and had only been described in pathogenic and non-pathogenic *X*. *campestris* (*Xc*) [37,40]. This MCP has been proved to restrict the host range of *Xc* to bean, garden stock and tomato [40] and a similar role could have on *Xap*. In addition, *Xap* also possess the MCP XCC0276 that had just been found in *X*. *campestris* [37].

STCRs are able to sense both extracellular and intracellular signals and have been also described in *Xcc* to participate in several processes, many of them related with biofilm formation, chemotaxis and motility [35,41–43]. *Xap* and *Xcc* showed similar SCTR patterns but some differences were revealed between them that might be related to the specific interaction with the host and its selection.

To determine which of the environmental sensors described above might play the major role in defining the host range, similarity matrices based on the sensor presence/absence were calculated. The lowest similarity between *Xcc* and *Xap* resulted from TBDTs profiles, indicating a possible source of specialization on them as a main character in sensing specific signal to the host in the infection process. This would be supported by the presence of conserved genes within the same species that would consist the core for TBDT within it.

Both species, *Xcc* and *Xap*, have the systems and factors required to survive and colonize plants, like flagella, exopolysaccharide, motility and biofilm formation etc. However, the variation in the environmental sensors will lead a variable response in different plant hosts; even if both species are able to survive on plant surfaces, the resulting combination of several sensors, in different strains, may induce different responses, as occur during biofilm formation on both LB and XVM2 or on host and non-host leaf surfaces. Higher biofilm formation on LB media by *Xap* indicates better aggregation when bacterial population increases. *Xap* is a real epiphytic bacteria that grow well on leaf surface, meanwhile for *Xcc* plant surface is a hostile environment where the bacteria show a restricted growth. The different biofilm abilities in high and low nutrient content media is concurrent with their different lifestyle, being *Xap* a good epiphytic bacteria compared to *Xcc* which is more efficient colonizing the apoplastic space than the leaf surface [22].

As previously described, xanthomonads harbour a large repertoire of cell-wall degrading enzymes which are secreted by T2SS, showing disparities within the genus or among strains that infects specific tissues into the same plant (as the case of pathovars *oryzae* and *oryzicola* of *X*. *oryzae*) [15,44]. According to the comparative analyses performed here, not remarkable variants were found in the case of the composition of T2SS, but interesting disparities were found between both species in the repertoire of the potential cell-wall degrading enzymes. *Xap* and *Xcc* may enclose different mechanisms to obtain nutrients, the first possess a higher number of enzymes because looks like it relies on those enzymes to break the cell and obtain nutrients [8,45,46], however in *Xcc* PthA effector contributes to break the plant cell and those cell wall degrading enzymes are used to further degrade the components released into the apoplast [47,48]. Mutants in *Xcc* affected in the T2SS, with cellulose activity truncated, only show a slight delay of symptoms after leaf inoculation [47]. All these evidences on cell wall degrading enzymes suggest their different role in *Xcc* and *Xap*. However, further functional analysis of the specific genes identified here for each species are required in order to determine if these variants reflects the preference of each species for their specific plant host as has been established in other phytopathogenic organisms [49].

In addition to the genes that could lead to a specific adaptation to their respective hosts in *Xap* and *Xcc*, previous comparative studies in *Xanthomonas* have identified the repertoire of T3Es as the major factor that contribute to virulence and host specificity [13,50]. In *Xanthomonas* the core set of T3Es seems to be conformed by seven effectors (AvrBs2, XopK, XopL, XopQ, XopR, XopX and XopZ). In our analyses this set of genes was found in most of the strains of *Xcc* but not in all of them. On the other hand, when searching these effectors in *Xap* (by sequence similarity with the genes of the list available at S2 Table), only five effectors of the core set were found with coverage/identity over the selected cutoff (80%). Meanwhile, homologous sequences to *xopK* (XAC3085) and *xopQ* (XAC4333) were found in every strain analyzed but their identity was lower than 80%. When comparing the sequences of these two effectors to the sequence of other *Xanthomonas* species, such as *X*. *campestris* ATCC 33913, they showed a coverage/identity over 90%. Therefore, it could be possible that these two effectors are actually in *Xap* but they showed dissimilarities to those found in *Xcc* due to their divergent evolutionary history [51]. Functional analysis are mandatory in order to determine if these two effectors, as well as the other effectors of *Xap* with similarity values under the cutoff selected, are operational T3Es. Recently, it has been described that the core set of T3Es of the three main pathogenic pathovars of *X*. *arboricola* is constituted by ten genes and in this study it was possible to find all of them with the exception of *xopAV* [51]. Additionally, in their recent analyses of the potential set of T3Es present in *X*. *arboricola* based on a machine-learning approach, up to 57 predicted T3Es were predicted for the pathogenic strains of *X*. *arboricola* therefore [51], it is possible for that reason, that the number of effectors described here could be underestimated. Further new analysis based in this novel approach, as well as functional or transcriptomic assays, could complement the work performed here to elucidate the real role of all these potential effectors in the host-pathogen interactions.

It is important to note that our study have permitted to find a group of T3Es that differentiate *Xap* and *Xcc* that could be of great interest in order to study their adaptation to different host plants. This is the case of those considered as TAL effectors (*avBrs3*/*pthA*, *pthA2*, *pthA3*, *pthA4*), as well as those non-TAL effectors *xopE1*, *xopA*, *xopAD*, *xopAE*, *xopAG*, *xopAP*, *xopAU*, *xopC1*, *xopI*, *xopP* found only in *Xcc* and *avrXccA2* that is present only in *Xap*. Currently, it is necessary to go beyond in the knowledge about TAL effectors in *Xap*. All genome sequences for *Xap* have been obtained by next generation sequencing, which is generally unable to identify this type of effectors. Consequently, to complete these genomes by resequencing the studied strains with other approaches that permitted to obtain longer reads, such as PacBio technology, is mandatory in order to determine the presence and variability of TAL effectors in *Xap* as has been demonstrated in other xanthomonads [52].

The comparative genomic work presented here gives some clues of the mechanisms that may play a role in the interaction of two important *Xanthomonas* with their hosts. However, in order to boost the knowledge related to the dynamics of its genome and to refine the understanding of its host specificity, it is mandatory to go further, improving the quality of the genomes, performing precise transcriptomic analyses, and more important, supporting the “omic” results by definitive functional studies to demonstrate the current mechanism of each of the genes involved in infection processes and host range.

## Material and methods

### Bacterial strains and growth conditions

*Xanthomonas* strains used in this study are listed in Table 1 and were routinely cultured at 27°C for 48 h on Luria Bertani (LB) medium (1.5% agar) or in LB broth (10 g l ^-1^ tryptone, 5 g l^-1^ yeast extract and 5 g l^-1^ sodium chloride). *X*. *arboricola* pv. *pruni s*trains CITA 9 and CITA 99 (= IVIA 3161-2-1 = CFBP 7100) were isolated from symptomatic leaves of *Prunus persica* cv. Merrill O’Henry and from *Prunus amygdalus* cv. Rumbeta, respectively, and their whole genome sequenced. These two bacterial strains are available and conserved in the collections of Centro de Investigación y Tecnología de Aragón (CITA, Aragón, Spain) and Instituto Valenciano de Investigaciones Agrarias (IVIA, Valencia, Spain). Genomic data from the remaining xanthomonads have been previously published [7,8,10,18,53] and are available in public databases PATRIC 3.5.27 (www.patricbrc.org) and NCBI (www.ncbi.nlm.nih.gov).

### Whole genome sequencing and genome comparative analyses

DNA from strains CITA 9 and CITA 99 was obtained from 30 ml of pure and fresh bacterial culture by using QIAamp DNA minikit (Qiagen, Barcelona, Spain). DNA quality and quantity were determined by electrophoresis in 1% agarose gel and by using the Qubit fluorometer (Invitrogen) according to the manufacturer instructions. Two samples of 73.6 ng μl^-1^ (CITA 9) and 71.8 ng μl^-1^ (CITA 99) of DNA were submitted for sequencing at STAB VIDA Next Generation Sequencing Laboratory (Caparica, Portugal), with the Illumina HiSeq platform, using 100 bp paired-end sequencing reads.

Quality of the obtained reads was assessed with the CLC Genomics Workbench (CLC v. 9.0.1) and only those reads with up to two ambiguous nucleotides, an error probability of less than 0.01, and with a minimum length of 30, were maintained for genome assembly. *De novo* assembly was performed with CLC and MIRA 4.0 and the assembly data produced by both software was merged with Sequencher 5.4 taking into account only those contigs with a lenght of at least 2,000 bp. Contigs with a minimum overlap of 100 bp and a minimum match percentage of 99% were merged and those contigs that were exclusively present in one of the assemblies were discarded. Finally, the high quality reads were mapped against the merged contigs to determine if the alignments were accurate and to manually correct possible misassembles. Automatic annotation of the draft genomes was conducted with the NCBI Prokaryotic Genome Annotation Pipeline [54] and with the Prokka annotation pipeline [55] using a specific database constructed with all the reference complete genome sequences of *Xanthomonas* available at NCBI's database. Output results from the Prokka pipeline (faa, fsa and gff files) were utilized as input data in order to determine similarities in the coding sequences (CDS) among all the genomes analyzed, as well as to calculate their average nucleotide identity based on the blast algorithm (ANI). The genome comparative analyses was performed with the Roary v. 3.11.2 [56], the Micropan v. 1.2 [57] and the Pyani v. 0.2.7 (available at https://github.com/widdowquinn/pyani) pipelines. The assignment of species-specific CDS to each cluster orthologous group (COG) was performed with the conserved domain database at NCBI using an expected value threshold of 0.001 [58].

Additionally, variation among the studied bacterial strains was assessed in respect of the presence of 410 genes with a putative or with known pathogenicity function previously reported in *Xanthomonas* (S2 Table) [12,16,21,31,37,59–73]. These genes encompassed environmental sensors (TonB- dependent transporters -TBDTs-, sensors of the two-component regulatory system -STCRs- and methyl accepting chemotaxis proteins -MCPs-), adherence and motility genes (structure and regulation of flagella, type IV pilus and non-fimbrial adhesins), production of xanthan gum, quorum sensing, cell wall degrading enzymes, secretion systems (types II, III and IV) as well as their associated effectors. To determine the presence/absence of these genes, they were searched with BLASTp by using an identity and coverage cutoff of 80%. Nucleotide sequences of the CITA 9 and CITA 99 genomes are available from the GenBank, DDBJ and EMBL databases (accession numbers: RWYS00000000 and RWYT00000000).

### Cluster analysis for environmental sensor profiles

Profiles of environmental sensors were obtained for each strain. Presence/absence of TBDTs, STCRs and MCPs were converted to a binary form and strains pairwise similarity was calculated according to the Jaccard´s coefficient and subjected to Unweigh Pair Group Method (UPGMA) cluster analysis. All the previous was computed using NTSYS 2.11T (Exeter Software, Setauket, NY).

### Biofilms on inert surface

Bacterial adhesion and biofilm formation were measured by a polypropylene 96-well plate assay as previously described [25,74]. Suspensions of strains *Xcc* 306 and CITA 33 in logarithmic growth phase, after incubation in LB, were washed twice and inoculated into LB or XMV2 culture media (20 mM NaCl, 10 mM (NH_4_)_2_SO_4_, 5 mM MgSO_4_ 7H_2_O, 1 mM CaCl_2_, 0.16 mM KH_2_PO_4_, 0.32 mM K_2_HPO_4_, 0.01 mM FeSO_4_, 10 mM fructose, 10 mM sucrose, 0.03% casein hydrolysed) onto poplypropylene 96-well plates. LB was used as a medium with high nutrient content and XVM2 as a medium that mimics apoplastic condition and induces the expression of virulence-associated genes [75,76].

Adhesion to the well surface was estimated after static bacterial growth in 200 μl of LB or XVM2 media for 72 h at 27°C. After this time, the medium was removed, and the microplates were incubated for an additional 72 h. Biofilm formation was measured after rinsing the plates with sterile distilled water (SDW) and staining with 0.3% crystal violet (CV) for 15 minutes. Excess stain was removed by rinsing the plates with SDW. Residual CV was solubilized by the addition of 200μL of 20:80 acetone:ethanol to each well and the absorbance quantified using a microplate reader set at A570 nm wavelength. Absorbance values for each strain were calculated as the means of three readings of five wells from three different assays. Means comparisons were performed by analysis of variance (ANOVA) and separated by Student-Newman-Keuls (SNK) multiple range test using Statgraphics Plus for Windows 4.1 (Statistical Graphics, Rockville, MD).

### Biofilms on biotic surface

The same *Xanthomonas* strains used above were used to evaluate biofilm formation on biotic surfaces. For inoculation, 30 ml of overnight LB cultures at 27°C were centrifuged 15 minutes at 1,400 g and washed twice with 10 mM MgCl_2_. A final concentration of 10^8^ cfu ml^-1^ achieved by adjusting absorbance to 0.1 was used for inoculations that were performed on 24 h detached fully expanded leaves of *Citrus x limon* (L.) Burm. f. (pro. sp.) and *Prunus dulcis* (Mill.) D. A. Webb. Plant leaves were superficially sterilized by immersion in 70% ethanol for 1 minute, 30 seconds in 0.05% sodium hypochlorite and thereafter washed three times with SDW. Leaf disks of 120 mm diameter were placed on 1.5% bacteriological agar plates and inoculated by adding 20 μl of 10^8^ cfu ml^-1^ bacterial suspension. 10 mM MgCl_2_ was used as negative control. The plates were incubated at 27°C using a light/dark photoperiod of 16/8 hours during 4 and 7 days. After incubation, non-attached bacterial population was washed with 0.1 M KPO_4_ buffer and bacterial aggregates fixed overnight at 4°C in 3% glutaraldehyde in KPO_4_ buffer. The post fixed treatment was performed in 2% of OsO_4_ in 1 M KPO_4_ buffer. After washing twice in 0.1 M KPO_4_ buffer and twice in SDW, samples were dehydrated in an ethanol series and critical point dried with CO_2_. The samples were coated with gold-palladium and observed in a Jeol JSM 6400 scanning electron microscope at the Electron Microscopy National Center of Complutense University of Madrid (UCM). Each combination plant-strain combination was performed in two discs and two plates and the assay was conducted twice.

### Bacterial persistence on biotic surface

Bacterial survival was evaluated on leaf discs by colony counting. Bacteria attached to the leave surface were harvested by shaking individually each plant sample in 1 ml SDW for 30 minutes. Serial dilutions from 10^−1^ to 10^−6^ were prepared and three drops of 20 μl of each dilution were plated onto two LB agar plates and incubated at 27°C for 48 h. Colony forming units (cfu) in each drop were counted and the average cfu ml^-1^ was calculated and used to quantify the viable and culturable population.

## Supporting information

S1 Table Pairwise average nucleotide identity (ANI) comparison among the studied strains of *Xanthomonas arboricola* pv. *pruni* (*Xap*) and *Xanthomonas citri* subsp. *citri* (*Xcc*). (XLSX)

S2 Table Presence of virulence-associated genes in *Xanthomonas arboricola* pv. *pruni* (*Xap*, 7 strains) and *Xanthomonas citri* subsp. *citri* (*Xcc*, 43 strians). (XLSX)
